# Markov modelling of viral load adjusting for CD4 orthogonal variable and multivariate conditional autoregressive mapping of the HIV immunological outcomes among ART patients in Zimbabwe

**DOI:** 10.1186/s12976-021-00145-y

**Published:** 2021-08-21

**Authors:** Zvifadzo Matsena Zingoni, Tobias F. Chirwa, Jim Todd, Eustasius Musenge

**Affiliations:** 1 Division of Epidemiology and Biostatistics, School of Public Health, Faculty of Health Sciences, University of the Witwatersrand, Johannesburg, South Africa; 2 Department of Population Health, London School of Hygiene and Tropical Medicine, London, UK

**Keywords:** CD4 counts, Viral load, Joint multistate models, Joint spatial maps, Multivariate conditional autoregressive prior, HIV disease progression

## Abstract

**Background:**

This study aimed to jointly model HIV disease progression patterns based on viral load (VL) among adult ART patients adjusting for the time-varying “incremental transients states” variable, and the CD4 cell counts orthogonal variable in a single 5-stage time-homogenous multistate Markov model. We further jointly mapped the relative risks of HIV disease progression outcomes (detectable VL (VL ≥ 50copies/uL) and immune deterioration (CD4 < 350cells/uL) at the last observed visit) conditional not to have died or become loss to follow-up (LTFU).

**Methods:**

Secondary data analysis of individual-level patients on ART was performed. Adjusted transition intensities, hazard ratios (HR) and regression coefficients were estimated from the joint multistate model of VL and CD4 cell counts. The mortality and LTFU transition rates defined the extent of patients’ retention in care. Joint mapping of HIV disease progression outcomes after ART initiation was done using the Bayesian intrinsic Multivariate Conditional Autoregressive prior model.

**Results:**

The viral rebound from the undetectable state was 1.78times more likely compared to viral suppression among patients with VL ranging from 50-1000copies/uL. Patients with CD4 cell counts lower than expected had a higher risk of viral increase above 1000copies/uL and death if their VL was above 1000copies/uL (state 2 to 3 (*λ*_23_): HR = 1.83 and (*λ*_34_): HR = 1.42 respectively). Regarding the time-varying effects of CD4 cell counts on the VL transition rates, as the VL increased, (*λ*_12_ and *λ*_23_) the transition rates increased with a decrease in the CD4 cell counts over time. Regardless of the individual’s VL, the transition rates to become LTFU decreased with a decrease in CD4 cell counts. We observed a strong shared geographical pattern of 66% spatial correlation between the relative risks of detectable VL and immune deterioration after ART initiation, mainly in Matabeleland North.

**Conclusion:**

With high rates of viral rebound, interventions which encourage ART adherence and continual educational support on the barriers to ART uptake are crucial to achieve and sustain viral suppression to undetectable levels. Area-specific interventions which focus on early ART screening through self-testing, behavioural change campaigns and social support strategies should be strengthened in heavily burdened regions to sustain the undetectable VL. Sustaining undetectable VL lowers HIV transmission in the general population and this is a step towards achieving zero HIV incidences by 2030.

**Supplementary Information:**

The online version contains supplementary material available at 10.1186/s12976-021-00145-y.

## Background

Antiretroviral therapy (ART) has since been the backbone of HIV prevention and control as it reduces viral load (VL) replication in the human host by blocking the virus life cycle [1]. Once the virus replication has been inhibited, the CD4 cell counts increase, and the individual life expectancy is expanded [2]. HIV patients’ management involves monitoring VL and CD4 cell counts prognostic markers through laboratory repeated measurements. The immunological markers can be utilized in understanding the HIV disease progression patterns among ART patients [2].

Multistate Markov models are mathematical models which have been used to evaluate HIV disease processes; however, the number of states, state cuts-off points and the number of transitions vary across studies [3, 4]. This explains the flexibility of the multistate models’ implementation, but the models become complex as the number of states and transitions increase. These models have been used extensively in HIV disease progression using CD4 cell counts states [5, 6] or VL states [7, 8] to define the model states (categories) separately. Of these two prognostic markers, VL is the preferred marker in HIV monitoring due to its high sensitivity. However, there had been a delay in the implementation and rolling out of VL testing in most developing countries due to cost-related challenges [9].

The low-middle-income countries (LMIC) have traditionally relied on the use of CD4 cell counts in HIV disease progression monitoring as this has been the readily available laboratory marker; however, if both VL and CD4 cell counts are available, it is imperative to model these prognostic markers jointly to understand better the HIV disease progression patterns. Joint modelling of these two prognostic markers helps to explain those effects which one marker cannot explain in the absence of the other marker.

In HIV disease monitoring programmes, Bayesian spatial modelling is an emerging tool to analyze spatially related multidimensional data with an underlying spatial process to guide policy [8, 10]; however, joint spatial modelling using the Bayesian intrinsic Multivariate Conditional Autoregressive (MCAR) prior has not been fully utilized in this field. The advantage of joint mapping is that it gives an understanding of the HIV dynamics and the spatial overlap between the joint mapped outcomes.

The main objective of our study was to jointly model HIV disease progression using two 5-stage time-homogenous multistate Markov models based on CD4 cell counts and VL. We firstly fitted two time-homogeneous multistate Markov models with states defined by CD4 cell counts and VL. In each multistate model, we jointly model these prognostic markers with one marker defining the finite multistate model states and the other marker forming the covariate matrix components of the regression model. The first covariate was an orthogonal variable generated using the principal component analysis (PCA) [11]. The second covariate was the time-varying “incremental transient states” variable to estimate the changes in transition rates over time with respect to how the marker changes [7]. Uniquely to this study is the inclusion of these two covariates in a single multistate model covariate matrix which has been a gap in previous studies that incorporated either the orthogonal variable or the time-varying covariate only [11]. We further performed a joint spatial mapping of HIV disease progression outcomes (detectable VL (VL ≥ 50copies/uL) and immune deterioration (CD4 < 350cells/uL) at the last observed visit conditional not to have died or become loss to follow-up (LTFU) to describe the spatial overlap of the two outcomes.

## Methods

### Data source, description and study design

This study was a secondary analysis of data from patients’ records compiled for monitoring and guiding programme planning. The data used in this study came from the Zimbabwe National ART programme collected through the electronic patients’ management system (ePMS) database described elsewhere [12, 13]. The electronic system was implemented nationally to improve and increase efficiency in HIV patients’ management and monitor their response to ART. A stratified sampling of health facilities offering HIV services was done; hence, a representative sample of primary, secondary, tertiary and quaternary health facilities which provide HIV services was achieved during the ePMS roll out. However, a continual up-scale is currently ongoing, which will ensure that the system covers all health facilities.

We considered individuals aged 15 years and above, with at least two repeated measurements of both CD4 cell counts and VL who initiated ART between 2004 and 2017. However, since this was programme data, the follow-up visits of the patients were intermittent, and there may have been clinical reasons for requesting CD4 count and VL measurements from patients. Each individual had an average follow-up period of 2 years, and the majority of the patients (79.2%) in the final sample considered for analysis were enrolled on ART between 2009 and 2015.

### The time-homogeneous Markov multistate model formulation

**Fig. 1:**
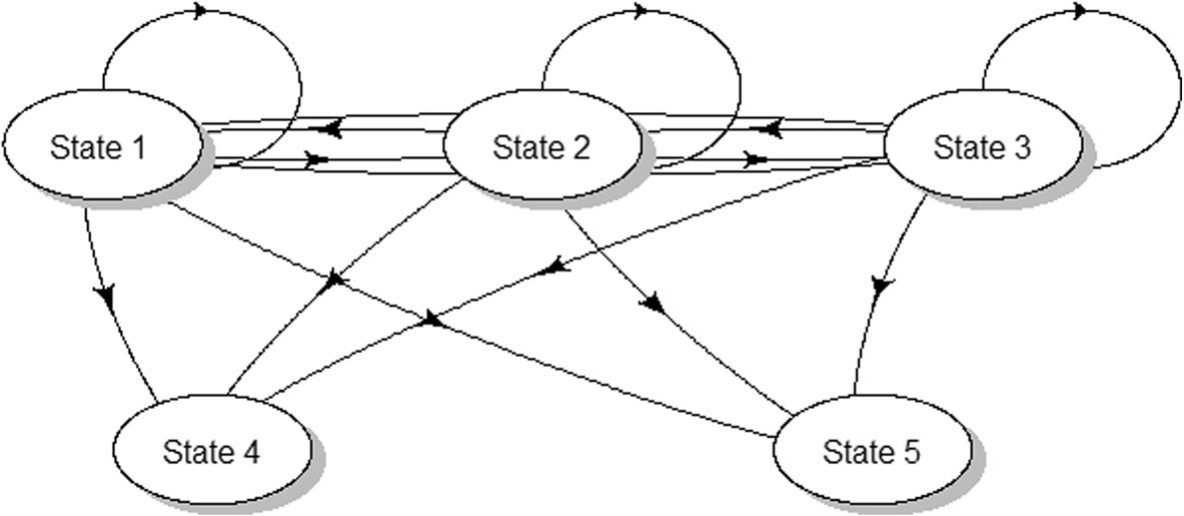
The schematic diagram for the possible transition between the defined state for both the viral load and the CD4 cell counts models **[**State 1(VL < 50or CD4 ≥ 500), State 2(50 ≤ VL < 1000 or 350 ≤ CD4 < 500), State 3(VL ≥ 1000 or CD4 < 350), State 4 (dead) and State 5 (Loss to follow-up (LTFU)**]**

A multistate model is a stochastic continuous-time process {*X*(*t*), *t* ∈ [0, *T*)years} defined as a finite space *X* = {1, 2, 3, 4, 5} based on VL or CD4 cell counts states:

1 $$ \mathrm{Viral}\ \mathrm{load}\ \left(\mathrm{VL}\right)\ \mathrm{states}\ \left(\mathrm{copies}/\mathrm{uL}\right)=\left\{\begin{array}{l}1;\mathrm{VL}<50\\ {}2;50\le \mathrm{VL}<1000\\ {}3;\mathrm{VL}\ge 1000\\ {}4;\mathrm{Dead}\\ {}5;\mathrm{Loss}\ \mathrm{to}\ \mathrm{follow}\hbox{-} \mathrm{up}\end{array}\right. $$

2 $$ \mathrm{CD}4\ \mathrm{cell}\ \mathrm{counts}\ \mathrm{states}\ \left(\mathrm{cells}/\mathrm{uL}\right)=\left\{\begin{array}{l}1;\mathrm{CD}4\ge 500\\ {}2;350\le \mathrm{CD}4<500\\ {}3;\mathrm{CD}4<350\ \\ {}4;\mathrm{Dead}\\ {}5;\mathrm{Loss}\ \mathrm{to}\ \mathrm{follow}\hbox{-} \mathrm{up}\end{array}\right. $$

where LTFU was defined as a failure of a patient to report for drug refill for at least 90 days from the last appointment date or if the patient missed the next scheduled visit date and never showed up again. The schematic presentation of the 5-stage multistate model is shown in Fig. 1. Based on these possible transitions shown in Fig. 1, the corresponding transition rates are defined by a 5 × 5 transition matrix *Q*(*t*) with *λ*_*ij*_ elements defining the movement between state *i* and state *j* with properties ${\sum}_{j=1}^5{\lambda}_{ij}=0$ and *λ*_*ii*_ =  − ∑_*i* ≠ *j*_*λ*_*ij*_. In reality, the individuals who become LTFU can return to the clinic for continual monitoring. However, in this study, we could not ascertain any returns of these participants after they became LTFU. This means the *λ*_5*j*_ transition rates from the LTFU state were not estimated as the data could not support this.

$$ Q(t)=\left(\begin{array}{ccccc}-\left({\lambda}_{12}+{\lambda}_{13}+{\lambda}_{14}+{\lambda}_{15}\right)& {\lambda}_{12}& {\lambda}_{13}& {\lambda}_{14}& {\lambda}_{15}\\ {}{\lambda}_{21}& -\left({\lambda}_{21}+{\lambda}_{23}+{\lambda}_{24}+{\lambda}_{25}\right)& {\lambda}_{23}& {\lambda}_{24}& {\lambda}_{25}\\ {}{\lambda}_{31}& {\lambda}_{32}& -\left({\lambda}_{31}+{\lambda}_{32}+{\lambda}_{34}+{\lambda}_{35}\right)& {\lambda}_{34}& {\lambda}_{35}\\ {}0& 0& 0& 0& 0\\ {}{\lambda}_{51}& {\lambda}_{52}& {\lambda}_{53}& {\lambda}_{54}& -\left({\lambda}_{51}+{\lambda}_{52}+{\lambda}_{53}+{\lambda}_{54}\right)\end{array}\right) $$

The effects of the covariates on each model were modelled using the semi-parametric proportionality hazards. We aimed to describe how the uncorrelated factor of VL values can explain the component of mortality, LTFU or HIV disease progression transition rates that cannot be explained by the CD4 cell counts alone ignoring VL measurements. To achieve this, we generated an orthogonal variable through the PCA technique, which is a data reduction approach used to combine highly correlated variables into uncorrelated components to improve model efficiency as described elsewhere [11]. The orthogonal variable was then included as a covariate in the proportionality hazard model. The second covariate was the time-varying VL measurements variable which was categorized into “incremental transient states”. The cut-off points for the time-varying VL covariate were similar to those defined in equation [2] above, excluding the mortality and the LTFU states. We simultaneously modelled the effects of these two covariates in a single model.

Therefore, we fitted two proportional hazard multistate models of the generic form:

3 $$ {\lambda}_{ij,k}/\boldsymbol{Z}(t)={\lambda}_{ij,(0)}\exp \left({\beta}_{ij}^{\prime }{\boldsymbol{Z}}_k\right),i\ne j $$

where *λ*_*ij*, *k*_/***Z***(*t*) is the transition rate between state *i* and state *j* at the time *t* given a covariate matrix ***Z*** and *λ*_*ij*, (0)_ is the baseline hazard rate of the model. The two multistate Markov models fitted were:

4 $$ {\lambda}_{ij,k(CD4)}/\boldsymbol{Z}(t)={\lambda}_{ij\left( CD4,0\right)}\exp \left({\beta}_{ij, CD4(1)}{P}_{VL(k)}^{\ast }+{\beta}_{ij, CD4(2)}{VL}_{states,k}\right) $$

5 $$ {\lambda}_{ij,k(VL)}/\boldsymbol{Z}(t)={\lambda}_{ij\left( VL,0\right)}\exp \left({\beta}_{ij, VL(1)}{P}_{CD4(k)}^{\ast }+{\beta}_{ij, VL(2)} CD{4}_{states,k}\right) $$

where equation [4] defines the CD4 cell counts multistate Markov model with *λ*_*ij*(*CD*4, 0)_ as the baseline transition intensity for an individual *k* with positive residual values for the orthogonal effect of VL $\left({P}_{VL(k)}^{\ast }=0\right)$ and the time-varying VL transient levels (*VL*_*states*, *k*_ = 1(*VL* < 50 *copies*/*uL*)). Similarly, equation [5] defines the VL multistate Markov model with *λ*_*ij*(*VL*, 0)_ representing the baseline transition intensities for an individual *k* with positive residual values for the orthogonal effect CD4 cell counts $\left({P}_{CD4(k)}^{\ast }=0\right)$ and the time-varying CD4 cell counts levels (*CD*4_*states*, *k*_ = 1(*CD*4 ≥ 500 *cells*/*uL*)). The *β*_*ij*, (*CD*4)*d*_ and *β*_*ij*, (*VL*)*d*_ for *d* = 1, 2 on both the CD4 cell count model and the VL model are the log-linear effects (coefficients) of the corresponding orthogonal and time-varying covariates.

Since the analyses were purely based on existing modelling approached within the “msm” R library, additional covariates like baseline CD4 cell counts, baseline VL values, age, sex and WHO staging could be adjusted for in the model; however, in this study, we encountered a convergence warning as more covariates were added. When the multistate Markov model fails to converge, it means the optimization criterion could not converge to the maximum likelihood; hence, the standard errors of the estimated parameters were not calculated, and the confidence interval estimates were missing on the printout of results. This modelling challenge is normally faced if there is data sparsity in some cells within the multistate model. Usually, the scaling factor is increased to a higher value adjusted to normalize the likelihood and prevent overflow within the optimization process; however, in our case, this was not helpful.

The final model selection was based on the Akaike Information Criterion (AIC) values which are defined as AIC =  − 2(Log − likelihood) + 2*q*, where 2*q* represents the variance component, *q* is the number of parameters to be estimated in the fitted model and the bias is defined by −2(Log − likelihood). The better fitting model is the model with the lowest AIC value. The nested models were assessed using the likelihood ratio test (LRT) defined as:

6 $$ LRT=-2\times {\log}_e\left({L}_1\left(\hat{\phi}\right)/{L}_2\left(\hat{\phi}\right)\right) $$

where ${L}_1\left(\hat{\phi}\right)$ is the likelihood for the simple (unsaturated) model with few covariates and ${L}_2\left(\hat{\phi}\right)$ is the likelihood for the full (saturated) model with additional covariate(s). A significant *p*-value < 0.05 for the LRT leads to the rejection of the null hypothesis (simple model is better) in favour of the alternative hypothesis (full model is better). To assess how well the final selected models predict HIV disease, mortality and LTFU, percentage prevalence in each state were plotted to compare the observed and the expected frequencies.

### Joint mapping of HIV progression based on two prognostic markers

Furthermore, an intrinsic MCAR prior model by Besag et al. (1991) [14] was fitted to jointly map the relative risks of the two HIV disease progression immunological outcomes (immune deterioration (CD4 < 350cells/uL) and detectable VL (VL≥50copies/uL) at the last observed visit) and estimate their shared geographical pattern. The choice of the VL immunological outcome cut-off point was based on the global goal to achieve an undetectable VL to minimize viral transmission in the general population [15] while the choice of the CD4 cell counts immunological outcome cut-off point was based on earlier studies that have shown that individuals with CD4 cell counts of 200-350cells/uL immune deterioration patterns are not significantly different from those with CD4 cell counts less than 200cells/uL [5].

The MCAR model is a special type of Gaussian Markov random field prior models (GMRF) [14, 16]. Let the number of observed individuals with detectable VL (VL≥50copies/uL) be*Y*_*VL*, *r*_, and the number of observed individuals with immune deterioration (CD4 < 350cells/uL) be *Y*_*CD*4, *r*_ at the last observed visit as reported for the region *S*_*r*_, where the set of regions {*S*_*r*_}, *r* = 1, 2, 3, …, *R* represents a finite number of the areas partitioned from the entire study area. In this study, we considered partitioning the Zimbabwe country into 10 provinces, i.e. *R* = 10. The geospatial variations are observed through an aerial map partitioned based on administrative spatial units. For each region, the expected number of individuals for each prognostic marker outcome (*E*_*VL*, *r*_ and *E*_*CD*4, *r*_)was calculated using the following generic formula:

7 $$ {E}_{r,q}={n}_{r,q}{\overline{r}}_q\equiv {n}_{r,q}\left(\frac{\sum_r{y}_{r,q}}{\sum_r{n}_{r,q}}\right)\ \mathrm{for}\ \mathrm{r}=1,2,3,\dots, R\ \mathrm{and}\ \mathrm{q}= CD4, VL $$

where ${\overline{r}}_q$ is the overall detectable VL (VL ≥ 50copies/uL) or immune deterioration (CD4 < 350 cell/uL) rate in the whole study region, *n*_*r*, *q*_ is the at-risk population in the region *r* and *y*_*r*, *q*_ is the total counts of individuals observed in the region. This approach is commonly referred to as “internal standardization”. The observed frequencies can be considered as the random variables while the expected frequencies are thought of as fixed and known functions of the at-risk population *n*_*r*, *q*_ in the region *r*. In this study, we assumed that the observed count data follow a Poisson distribution for the two prognostic marker outcomes, i.e.

8 $$ {\displaystyle \begin{array}{l}{Y}_{q,r}\sim Poisson\left({E}_{q,r}{\theta}_{q,r}\right)\mathrm{for}\ \mathrm{r}=1,2,3,\dots, R\ \mathrm{and}\ \mathrm{q}= CD4, VL\\ {}\log \left({\theta}_{q,r}\right)=\log \left({E}_{q,r}\right)+{\alpha}_q+{S}_{q,r}\end{array}} $$

where *α*_*q*_ is an intercept term representing the baseline (log) relative risk of disease progression outcomes *q* across the study region. We further assumed that the log relative risks are spatially correlated across the regions, and the log relative risks for the two prognostic markers are also correlated within each region *r* due to shared region-level unmeasured risk factors. These assumptions were supported through the intrinsic bivariate CAR prior of a 2 × *R* dimensional matrix of *S*_*q*, *r*_ values. The spatial prior is expressed as:

9 $$ {S}_q\mid {S}_{1\left(-q\right)},{S}_{2\left(-q\right)}\sim \mathrm{Bivariate}\ \mathrm{Normal}\left({\overline{\boldsymbol{S}}}_q,\boldsymbol{V}/{n}_q\right) $$

where *S*_1(−*q*)_, *S*_2(−*q*)_ denotes the elements of the 2 × *R* matrix, ${\overline{\boldsymbol{S}}}_r=\left({\overline{S}}_{r,1},{\overline{S}}_{r,2}\right)$ and ${\overline{S}}_{r,p}={\sum}_{\mathrm{f}\ \mathrm{in}\ {\sigma}_r}{S}_{fp}/{n}_r$ where *σ*_*r*_ and *n*_*r*_ denote the set of labels of the “neighbours” of the region *r* and the number of neighbours, respectively assuming a *p* = 2. The matrix ***V*** is a 2 × 2 covariance matrix with diagonal elements *v*_11_ and *v*_22_ denoting the conditional variances of *S*_1_ and *S*_2_ respectively, and an off-diagonal element *v*_12_ denoting the conditional within-area covariance between *S*_1_ and *S*_2_.

The MCAR model was performed in the OpenBUGS, which is open-source statistical software and the code used is provided in the Additional file 1 section. We ran 10,000 Markov chain Monte Carlo (MCMC) simulations, burn-in of 2000 and thinning of 10. A prior sensitivity analysis was done through varying prior distributions and parameter values. We ran simultaneously two chains and the results reported are based on the better chain of initial values. Model diagnostics were assessed through trace plots which should traverse rapidly in the same region, density plots which should show a smooth curve and autocorrelation plots which should show a quick sharp drop in early lags.

## Results

A total of 3896 participants contributed 8655 follow-up observations. There were 2551(65.5%) females and 1345(34.52%) were males. The average age was 38.23 ± 11.37 years and 1388(35.6%) participants were aged 35–44 years. At baseline, the median CD4 cell count was 211cells/uL with an interquartile range (IQR) of 114-320cells/uL and 1846 (47.4%) participants had CD4 cell counts of below 350cells/uL. The median VL was 57copies/uL (IQR: 48-66copies/uL) and 2557 (65.6%) participants had VL between 50 and 1000 copies/uL at baseline. All participants were on three-drug combination therapy and none were receiving protease inhibitors (PI) or integrase inhibitors INSTI) regimen. Most of the participants (95%) were on a first-line three-drug combination therapy which was a combination therapy of two nucleoside reverse transcriptase inhibitors (NRTI) namely Tenofovir (TDF) and Lamivudine (3TC); and one non-NRTI namely Efavirenz (EFV), that is, TDF + 3TC + EFV, while 5% were on second-line drugs.

Table 1 shows the simple regression model results used to generate the orthogonal covariates for both the CD4 cell counts and the VL model. We observed a significant correlation between CD4 cell counts and VL for both models and both slopes were negative as expected.

**Table 1:** Estimated regression coefficients for the simple linear regression model for the viral load on CD4 cell counts and CD4 cell counts on viral load

	Parameter	Estimate	R-adjusted	P > |t|	VIF^a^
**Viral load on CD4 cell counts**	*β*_1_	333.0816 (321.51–344.66)	0.5134	< 0.001	2.29
	*β*_2_	−0.003955 (− 0.008 to − 0.0003)		0.035	
**CD4 cell counts on viral load**	*β*_1_	62.86 (5.19–120.52)	0.5646	0.033	1.03
	*β*_2_	−0.12 (− 0.21 to − 0.04)		0.006	

^a^variance inflation factor

Table 2 presents a detailed summary of the log-likelihood values, LRT statistics, LRT *p*-values and the AIC values. Using the LRT results for the nested models, we assessed a better fit model between the no covariate model $\left[{\lambda}_{ij}(t)={\lambda}_{ij(0)}\exp \left({\beta}_{ij}^{\prime}\right),i\ne j\right]$ and the orthogonal adjusted model [*λ*_*ij*_(*t*) = *λ*_*ij*(0)_ exp(*β*_*ij*_ × *P*^∗^)] for both the CD4 cell counts and VL multistate models.

**Table 2:** Model selection process for the various model fitted for both viral load and CD4 cell counts multistate models

Covariate	−2*LL^**a**^	AIC^**b**^	Models compared	LRT ^**c**^ statistic	Df^**d**^	***P***-value
**Viral load multistate model**						
*Model 1*- No covariates	7074.605		–	–	–	–
*Model 2*- CD4 effects	7014.406	7062.99	Model 1 vs Model 2	59.61457	12	2.65e-08^**^
*Model 3*- CD4 time-varying effects	7007.724	7055.72	Model 1 vs Model 3	66.88163	12	1.22e-09^**^
*Model 4*- CD4 effects+ CD4 time-varying effects	6930.714	7002.71	Model 2 vs Model 4	83.69604	12	8.10e-13^**^
			Model 3 vs Model 4	77.01372	12	1.53e-11^**^
**CD4 cell counts multistate model**						
*Model 1*- No covariates	7334.08		–	–	–	–
*Model 2*- VL effects	7298.644	7342.64	Model 1 vs Model 2	35.36379	11	2.16e-04^**^
*Model 3*- VL time-varying effects	7256.166	7344.17	Model 1 vs Model 3	77.84109	33	1.74e-05^**^
*Model 4*- VL effects + VLtime-varying effects	7227.461	7337.46	Model 2 vs Model 4	71.18325	33	1.28e-04^**^
			Model 3 vs Model 4	28.70595	11	2.52e-03^**^

^a^Log-likelihood ^b^Akaike’s information criterion ^c^Likelihood ratio test ^d^degrees of freedom ^**^Significant at 5%

Both the CD4 cell count and the VL multistate models showed that the model with the orthogonal variable (model 2) was a better fit compared to the no variate model (model 1). Similarly, the multistate models which adjusted for the time-varying effects of the other prognostic marker, [*λ*_*ij*, *VL*_(*t*) = *λ*_*ij*(0)_ exp(*β*_*ij*_ × *CD*4_*state*_)and *λ*_*ij*, *CD*4_(*t*) = *λ*_*ij*(0)_ exp(*β*_*ij*_ × *VL*_*state*_)] (model 3) were better fit compared to the no covariate models (model 1). Simultaneously adjusting for the orthogonal variable and the time-varying effects on both the CD4 cell count model and VL model (model 4) further improve the model, P-value< 0.05; hence, the interpretation of results was based on these models (model 4).

### Modelling of CD4 cell counts adjusting for the viral load orthogonal and time-varying effects

The orthogonal and the time-varying VL effects were regressed in a single model. In this model, the movement between state *i* to state *j* for *i* > *j* defines an increase in CD4 cell counts which indicates immune recovery process while *i* < *j* defines a decrease in CD4 cell counts, which means immune deterioration process.

In Table 3, results show that the rates of decrease in CD4 cell counts (immune deterioration) among patients in state 2 (CD4 cell count range of 350–500 cells/uL; *λ*_23_ = 0.0404) were 11.22 times higher than the rates of increase in CD4 cell counts (immune recovery) among patients in state 3 (CD4 < 350cells/uL; *λ*_32_ = 0.0036). Patients in state 2 (CD4 cell count range of 350–500 cells/uL) were more likely to become LTFU (*λ*_25_ = 0.0551). The positive VL log-linear effects showed an increased risk of immune deterioration for an individual in state 2 (CD4 cell count range of 350–500 cells/uL) to state 3 (CD4 < 350cells/uL) $\left({\beta}_{23}{P}_{VL(k)}^{\ast }=0.4762\right)$. Similarly, the log-linear effects of the time-varying VL variable showed an increased risk of immune deterioration for the *λ*_23_ transition (*β*_23_*VL*_*states*, *k*_ = 1.0791)**.**

**Table 3:** The joint effects of time-varying viral load and orthogonal viral load variables on the CD4 cell counts transition rates Markov model

Transition Rate *λ*_*ij*_	Baseline	Log-linear *β*_*ij*_		Hazard exp(*β*_*ij*_)		Time-varying viral load (VL) levels *VL*_*states*, *k*_		
	*λ*_*ij*(*CD*4, 0)_	*VL*_*states*, *k*_	${P}_{VL(k)}^{\ast }$	*VL*_*states*, *k*_	${P}_{VL(k)}^{\ast }$	State 1	State 2	State 3
State 1 to 2 *λ*_12_	0.0192	0.1678	0.1739	1.0827	1.1899	0.0182	0.0161	0.02763
State 1 to 3 *λ*_13_	0.2856	− 0.3505	−1.8298	0.7044	0.1604	0.7431	0.4755	1.3939
State 1 to 5 *λ*_15_	0.0381	0.5612	0.5131	1.7528	1.6704	0.0245	0.0333	0.0535
State 2 to 1 *λ*_21_	0.0114	− 0.6837	0.6186	0.5048	0.8563	0.0168	0.0068	0.0088
State 2 to 3 *λ*_23_	0.0404	0.4762	1.0791	1.6099	2.9419	0.0239	0.0261	0.0948
State 2 to 4 *λ*_24_	0.0086	− 1.0434	− 0.6853	0.3663	0.5039	0.0283	0.0067	0.0126
State 2 to 5 *λ*_25_	0.0551	0.5786	−0.0711	1.7836	0.9314	0.0338	0.0729	0.0416
State 3 to 1 *λ*_31_	0.0062	− 1.6657	− 0.5138	0.1891	0.5982	0.0235	0.0055	0.0077
State 3 to 2 *λ*_32_	0.0036	0.2016	−0.3475	1.2233	0.7064	0.0039	0.0032	0.0131
State 3 to 4 *λ*_34_	0.0079	0.1820	0.1034	1.1996	1.1089	0.0057	0.0069	0.0421
State 3 to 5 *λ*_35_	0.0195	0.2806	−0.1784	1.3239	0.8366	0.0156	0.0217	0.0305

model information: −2*log-likelihood = 7227.461; Akaike information criterion = 7337.461

The time-varying VL values and the negative VL (residuals covariate) had an increasing effect on the risk of death in this cohort for individuals with CD4 < 350cell/uL (hazard ratio (HR) =1.75 and HR = 1.67, respectively). Regarding the time-varying effects of VL on the CD4 cell counts transition rates, as the CD4 cell counts increased for an individual with a CD4 cell count range of 350–500 cells/uL (*λ*_23_), the transition rates increased with an increase in VL levels over time. Similarly, individuals with CD4 ≥ 500cells/uL (*λ*_15_) and those with CD4 < 350cell/uL (*λ*_35_) showed an increased risk of becoming LTFU as their VL increased over time. A comparable trend was observed for individuals with CD4 < 350cell/uL (*λ*_34_) who exhibited an increased risk of death with an increase in VL over time.

### Modelling of viral load adjusting for the CD4 cell counts orthogonal and time-varying effects

We fitted a time-homogeneous multistate Markov model to evaluate HIV disease progression based on VL defined states. The effects of the orthogonal and the time-varying CD4 cell counts effects were accounted for in a single model. In this model, the movement between state *i* to state *j* for *i* > *j* defines a VL suppression while *i* < *j* defines VL rebound.

In Table 4, the rates of viral rebound among patients with VL < 50copies/uL) (*λ*_12_ = 0.0286) were 1.78 times higher than the rates of VL suppression among patients with a VL range of 50-1000copies/uL (*λ*_21_ = 0.0161). However, patients with VL ≥ 1000copies/uL were 7.77 times more likely to die (*λ*_32_ = 0.0035 vs *λ*_34_ = 0.0272) and 9.8 times more likely to become LTFU (*λ*_32_ = 0.0035 vs *λ*_35_ = 0.0343). The time-varying CD4 cell counts had an increasing effect on the risk of viral increase from a VL range of 50-1000copies/uL to VL ≥ 1000copies/uL in this cohort, HR =1.23.

**Table 4:** The joint effects of time-varying CD4 cell counts levels and orthogonal CD4 cell counts variables on the viral load transition rates Markov model

Transition Rate *λ*_*ij*_	Baseline	Log-linear *β*_*ij*_		Hazard exp(*β*_*ij*_)		Time-varying CD4 cell counts levels *CD*4_*states*, *k*_		
	*λ*_*ij*(*VL*, 0)_	*CD*4_*states*, *k*_	${P}_{CD4(k)}^{\ast }$	*CD*4_*states*, *k*_	${P}_{CD4(k)}^{\ast }$	State 1	State 2	State 3
State 1 to 2 *λ*_12_	0.0286	0.0434	−0.3383	1.0443	0.713	0.0305	0.0321	0.0337
State 1 to 3 *λ*_13_	0.0025	− 1.0072	− 0.3336	0.3652	0.7163	0.0167	0.0059	0.0021
State 1 to 4 *λ*_14_	0.0082	0.0288	0.0247	1.0292	1.0243	0.0078	0.0081	0.0083
State 1 to 5 *λ*_15_	0.0159	− 0.7155	−1.1045	0.4889	0.3312	0.1774	0.0422	0.0206
State 2 to 1 *λ*_21_	0.0161	− 0.3926	− 0.2849	0.6762	0.7521	0.0351	0.0237	0.0161
State 2 to 3 *λ*_23_	0.0008	0.2065	0.6052	1.2293	1.8315	0.0004	0.0005	0.0006
State 2 to 4 *λ*_24_	0.0057	0.0725	−0.2381	1.0752	0.7881	0.0056	0.0061	0.0066
State 2 to 5 *λ*_25_	0.0305	− 0.7458	− 0.9312	0.4744	0.3941	0.3372	0.0759	0.0361
State 3 to 1 *λ*_31_	0.0266	− 0.9855	−0.7571	0.3733	0.4691	0.1926	0.0728	0.0275
State 3 to 2 *λ*_32_	0.0035	− 1.4092	−2.8306	0.2443	0.0589	0.1359	0.0324	0.0077
State 3 to 4 *λ*_34_	0.0272	− 0.0811	0.3475	0.9221	1.4155	0.0255	0.0237	0.0221
State 3 to 5 *λ*_35_	0.0343	− 0.5176	0.0947	0.5959	1.0993	0.1273	0.0457	0.0273

model information: −2*log-likelihood = 6930.71; Akaike’s information criterion = 7002.71

Similarly, the negative CD4 cell count residual was associated with an increased risk of viral increase from a VL range of 50-1000copies/uL to VL ≥ 1000copies/uL $\left({\beta}_{23}{P}_{CD4(k)}^{\ast }=0.6052;\mathrm{HR}=1.83\right)$; and an increased risk of death among individuals with VL ≥ 1000copies/uL $\left({\beta}_{34}{P}_{CD4(k)}^{\ast }=0.3475;\mathrm{HR}=1.42\right)$**.** Regarding the time-varying effects of CD4 cell counts on the VL transition rates, as the VL increased (*λ*_12_ and *λ*_23_) the transition rates increased with a decrease in the CD4 cell counts over time while as the VL decreased (*λ*_21_, *λ*_31_ and *λ*_32_) the transition rates decreased with a decrease in the CD4 cell counts over time. The mortality rates of individuals with VL < 50copies/uL and those with VL range between 50-1000copies/uL increased over time as CD4 cell count decreases (*λ*_14_ and *λ*_24_). Regardless of the individual’s VL state, the transition rates to become LTFU decreased with a decrease in CD4 cell counts. Individuals with high CD4 cell counts were more likely to become LTFU in this cohort (*λ*_15_, *λ*_25_ and *λ*_35_); however, patients with CD4 ≥ 500cells/uL and a VL range of 50-1000copies/uL had an increased risk of becoming LTFU.

### Multistate Markov models assessment

**Fig. 2:**
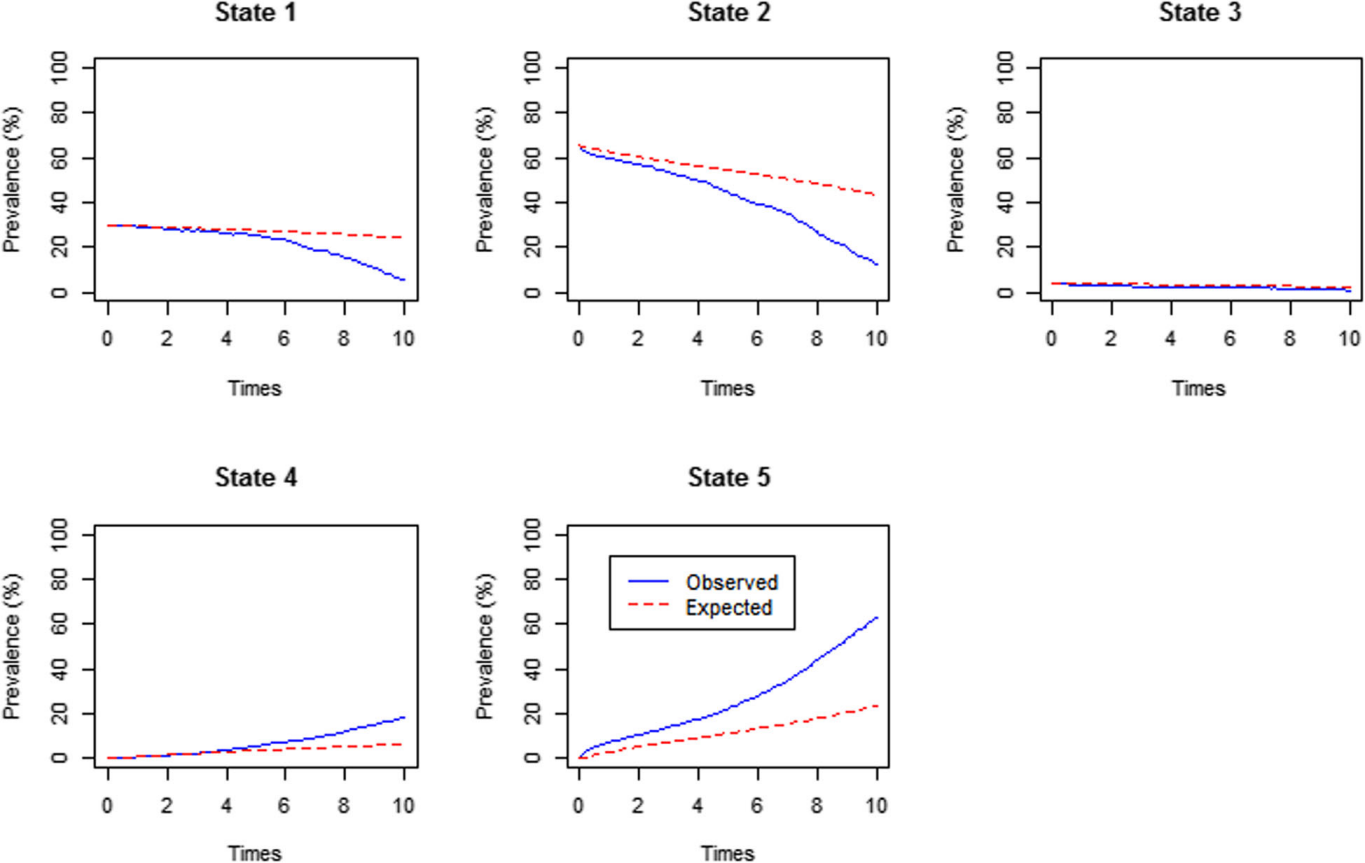
The percentage prevalence plot of orthogonal CD4 cell counts and the time-varying CD4 cell counts values on the viral load multistate model  [State 1(VL < 50), State 2 (50 ≤ VL < 1000), State 3(VL ≥ 1000), State 4 (dead) and State 5 (Loss to follow-up (LTFU)]

We performed a post-estimation test to assess which of the two multistate models fit better on this data in describing the HIV disease progression, mortality and becoming LTFU. We used the prevalence plot to compare the observed and the expected percentage prevalence for CD4 cell counts and VL multistate models. The VL multistate model showed a perfect fit for state 3(VL ≥ 1000copies/uL) and fair (moderate) fit for state 1 (VL < 50copies/uL) and state 4 (died). State 3 (VL ≥ 1000copies/uL) and state 5 (LTFU) of this model were not perfectly fitted by this model (Fig. 2).

**Fig. 3:**
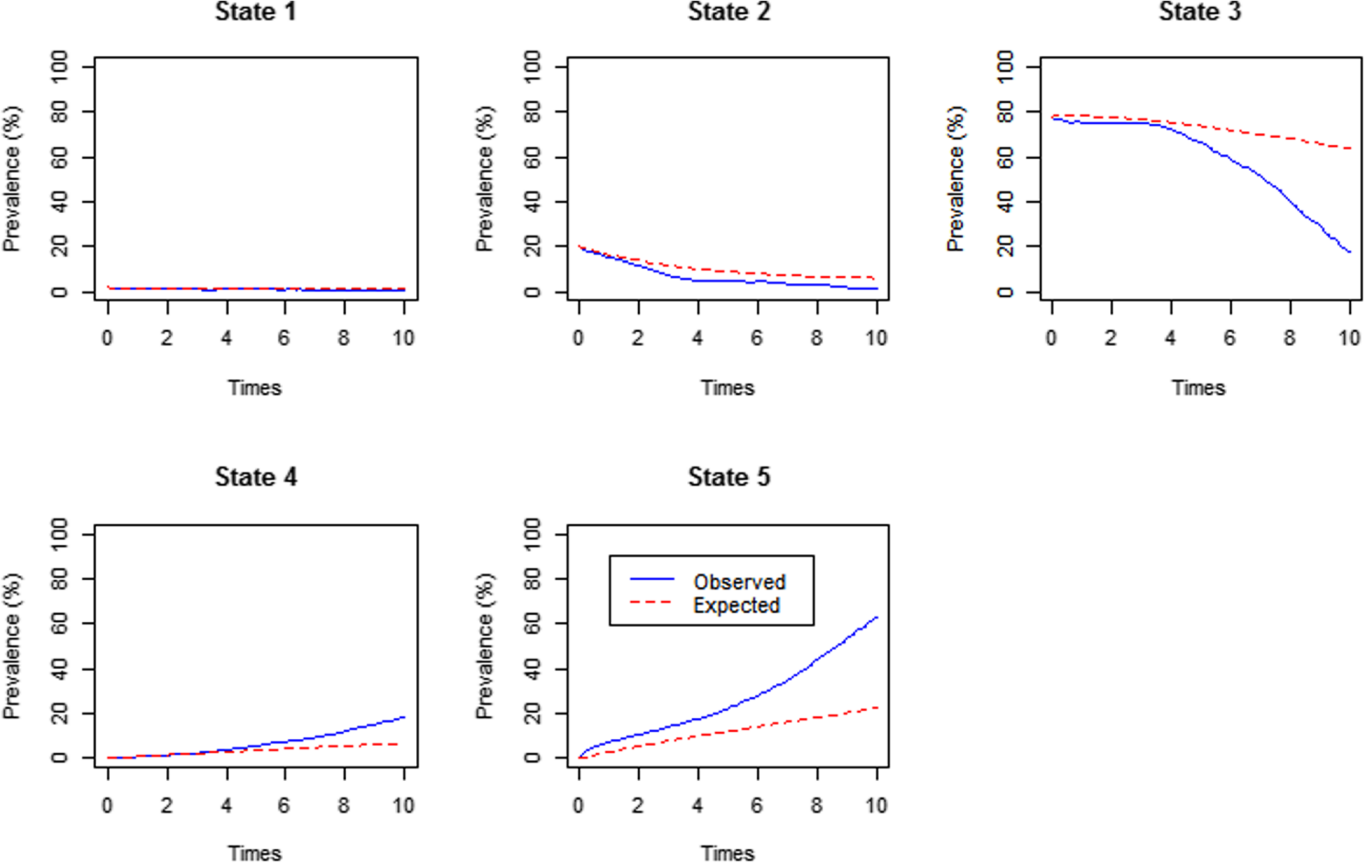
The percentage prevalence plot of orthogonal viral load variable and the time-varying viral load variables on the CD4 multistate model [State 1(CD4 ≥ 500), State 2 (350 ≤ CD4 < 500), State 3 (CD4 < 350), State 4 (dead) and State 5 (Loss to follow-up (LTFU)**]**

Regarding the CD4 cell counts multistate model, state 1 (CD4 ≥ 500cell/uL) was perfectly fitted while state 2 (350 ≤ CD4 < 500) and state 4 (died) were fairly fitted. State 3 (CD4 < 350cell/uL) was perfectly fitted between time 0 and time 5 years only while state 5 (LTFU) was not perfectly fitted (Fig. 3). Since the percentage prevalence plots could not give conclusive results on the better model and the LRT was not appropriate as the models were not nested, we used the AIC values. The VL multistate model had an AIC = 7002.71 while the CD4 cell counts multistate model had an AIC = 7337.46; hence, the VL multistate model was a better fit model.

### Sub-analysis of the spatial covariate (province of ART enrolment) on the multistate models and joint mapping of the two immunological HIV conditions using the multivariate intrinsic CAR model

We further assessed the effects of the region variable (province) on the VL and CD4 cell count multistate models to identify any association between the observed transition processes and the region. We estimated the HR summarised in Table 5. In general, we observed that there was an association between the transition intensities and the province administrative level. We found that 57.6% of the transition rates for CD4 cell count and VL models adjusting for province covariate showed the same effect direction (risk or protective effect) of the hazard ratios; however, there were variations in the magnitude of the risk of transitions among provinces. Immune deterioration (decrease in CD4 cell counts) was evident among patients belonging to Mashonaland East (P4) (state 1 to 3 and state 2 to 3) and Matabeleland North (P7) (state 1 to 2), while the immune recovery was most evident among patients from Masvingo (P6).

**Table 5:** The spatial effects (province-level) on the viral load and CD4 cell counts multistate model adjusted for time-varying effects and residual effect of the other prognostic marker with Harare province as a reference category

Province Hazard Ratio	Transition processes for Viral load (VL) and CD4 cell counts multistate models											
	State 1 to State 2	State 1 to State 3	State 1 to State 4	State 1 to State 5	State 2 to State 1	State 2 to State 3	State 2 to State 4	State 2 to State 5	State 3 to State 1	State 3 to State 2	State 3 to State 4	State 3 to State 5
P1: VL	0.004	0.405	**3.711**	0.311	0.327	**1.296**	**26.41**	0.089	0.042	0.162	0.047	0.089
CD4	0.912	0.854	____	0.794	**1.253**	0.996	**1.001**	0.972	**1.821**	**1.181**	**1.683**	**1.089**
P2: VL	0.056	0.514	0.001	0.109	**5.611**	**1.834**	0.021	0.721	**1.729**	**1.981**	0.022	0.071
CD4	**1.705**	0.615	____	**1.019**	0.831	**1.595**	0.866	0.614	0.899	0.561	0.347	0.151
P3: VL	0.342	0.313	**6.096**	0.001	0.885	**1.033**	0.778	0.206	0.301	0.704	0.003	0.007
CD4	0.958	**1.861**	_____	0.869	0.945	**1.364**	0.961	0.775	**1.493**	**1.204**	**1.469**	0.854
P4: VL	**3.191**	0.208	**5.524**	**2.625**	**3.933**	0.028	**3.066**	**2.082**	**1.041**	**2.447**	0.043	0.064
CD4	0.709	**9.371**	_____	**1.664**	0.732	**6.099**	**1.019**	**2.636**	**2.186**	**2.279**	**2.894**	**2.743**
P5: VL	0.002	0.411	2.101	**1.671**	0.172	0.974	0.347	**2.155**	0.537	0.925	0.013	**1.759**
CD4	**1**.**216**	**1.125**	_____	**1.159**	0.974	**1.058**	0.981	0.844	**1.358**	**1.459**	**1.114**	**2.543**
P6: VL	0.256	**1.633**	0.019	0.379	**9.378**	**2.479**	0.081	**3.455**	**2.525**	**9.423**	0.094	0.138
CD4	0.934	**1.263**	____	0.967	**1.747**	**3.451**	0.928	**1.046**	**6.262**	**5.288**	**1.241**	**1.439**
P7: VL	**1.198**	**16.68**	**2.564**	0.428	**3.145**	0.025	**2.984**	0.568	**9.323**	0.653	0.225	0.398
CD4	**1.744**	0.331	_____	0.712	**1.509**	0.269	**1.519**	0.479	0.439	0.851	**2.565**	0.585
P8: VL	0.013	0.507	**6.321**	0.834	0.414	**1.677**	0.701	0.133	0.705	0.924	0.455	0.029
CD4	0.968	**1.641**	_____	0.915	0.975	**2.006**	**1.229**	**1.047**	**1.711**	**1.489**	**1.766**	**1.231**
P9: VL	0.001	0.101	0.005	0.087	0.095	0.816	0.177	0.026	0.036	0.153	0.001	0.095
CD4	0.978	**1.249**	_____	0.934	0.907	0.652	0.932	0.953	0.618	0.764	0.654	0.355

Bold-faced values are hazard ratios above 1;

CD4 model information: −2*Log-likelihood = 6932.39; CD4 model AIC = 7196.39; Viral load model information: − 2*log-likelihood = 6429.69; Viral load model AIC = 6716.69;

P1 = Bulawayo, P2 = Manicaland, P3 = Mashonaland Central, P4 = Mashonaland East, P5 = Mashonaland West, P6 = Masvingo, P7 = Matabeleland North, P8 = Matabeleland South, P9 = Midland;

___indicates no observed transition for that particular multistate model

Becoming LTFU was observed to be highest among patients from Mashonaland East (P4) regardless of their CD4 cell count. We found that the risk of viral rebound to above 50copiels/uL was high among patients from Matabeleland North (P7) (state 1 to 2), Mashonaland East (P4) (state 1 to 3) regions. In contrast, viral suppression to undetectable levels (VL < 50copies/uL) was evident among patients from Masvingo (P6) (state 2 to 1) and Matabeleland North (P7) (state 3 to 1). The risk of becoming LTFU was high among patients from Mashonaland East (P4) if their VL < 50copiels/uL, Masvingo (P6) if their VL range was between 50-1000copies/uL and Mashonaland West (P5) if their VL ≥ 1000copies/uL.

To get a pictorial view of the spatial patterns and correlation between the CD4 cell counts marker and the VL measurements, we fitted the multivariate intrinsic CAR prior model with the province as the spatial unit. We jointly modelled those patients who had a VL ≥ 50copies /uL (VL state 2 and 3 combined) at the end of the follow-up to define that group that might not have attained undetectable VL or have a VL rebound to detectable levels, and those patients who had a CD4 < 350cells/uL (CD4 state 3) to define that group that is still in the immune-deterioration phase at the end of the follow-up period.

Table 6 shows the posterior estimates after the joint mapping of the two immunological outcomes for HIV disease progression among ART patients based on CD4 cell counts and VL. The posterior correlation between the spatially structured risk components of having a detectable VL (VL ≥ 50copies/uL) and immune deterioration (CD4 < 350cells/uL) was 61.3% (95% credible interval (CI): 47–97%). This strong correlation suggests strong shared geographical patterns of the risk of immune deterioration defined by these two prognostic markers. The baseline (log) relative risk of the HIV disease progression based on detectable VL (VL ≥ 50copies/uL) was estimated at − 0.472 while that of immune deterioration (CD4 < 350cells/uL) was − 0.043.

**Table 6:** The posterior estimates for the multivariate conditional autoregressive model for the two HIV disease progression prognostic markers

Parameter	Estimate	Standard deviation	95% Credible interval
Alpha [VL ≥ 50 copies/uL]	−0.472	0.0628	−0.60 to − 0.35
Alpha [CD4 < 350 cells /uL]	−0.043	0.0314	−0.11 to 0.02
Correlation	0.613	0.3815	0.47 to 0.97
Sigma [VL ≥ 50 copies/uL	1.226	0.3505	0.72 to 2.08
Sigma [CD4 < 350 cells /uL]	0.147	0.0668	0.06 to 0.31

**Fig. 4:**
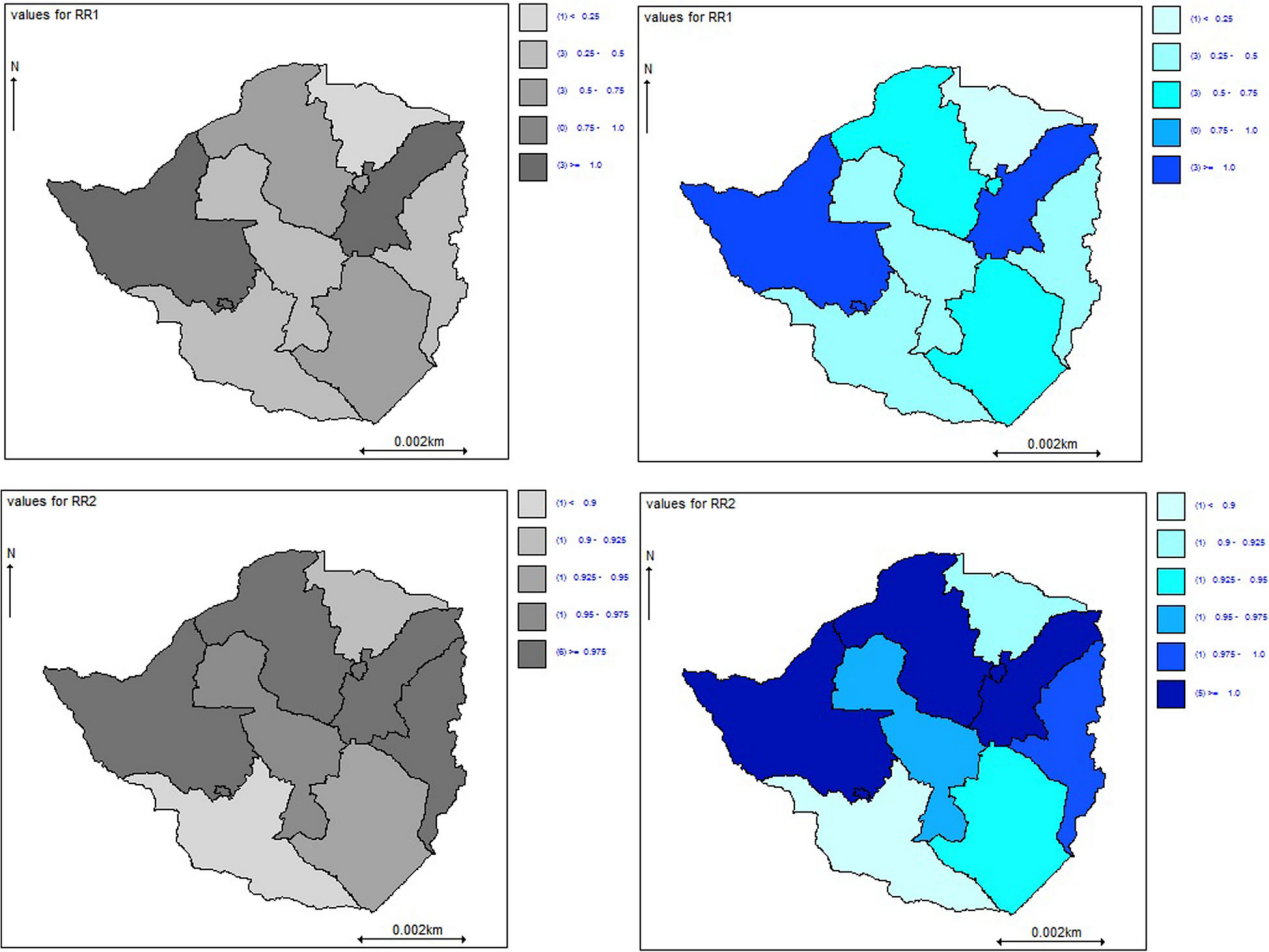
The joint mapping of the posterior relative risk of the HIV disease progression defined by the viral load (RR1) and CD4 cell counts (RR2) under the multivariate conditional autoregressive model. The maps were generated from OPENBUGS version 3.2.3 [17] https://www.mrc-bsu.cam.ac.uk/software/bugs/openbugs/

The joint mapping of the posterior relative risk of the HIV disease progression defined by the VL (RR1) and CD4 cell counts (RR2) under the MCAR model is shown in Fig. 4. The dark blue or deep grey colours indicate areas with high relative risk while the light blue or light grey colours show regions with the lowest relative risks of the defined conditions. These maps show a geographical overlap of detectable VL (VL ≥ 50copies/uL) and immune deterioration (CD4 < 350cells/uL) in seven provinces. Patients in Matabeleland North province bordering Botswana and Zambia; and Mashonaland East province toward the Mozambique border had higher relative risks of detectable VL (VL ≥ 50copies/uL), and immune deterioration (CD4 < 350cells/uL) outcomes in addition to Bulawayo and Harare metropolitan provinces.

## Discussion

The main objective of our study was to jointly model HIV disease progression using two 5-stage time-homogenous multistate Markov models based on CD4 cell counts and VL. The multistate models accounted for the orthogonal and the time-varying covariates simultaneously which has been a gap in previous studies. The spatial overlap of the HIV disease progression outcomes (detectable VL (VL ≥ 50copies/uL) and immune deterioration (CD4 < 350cells/uL) at the last observed visit conditional not to have died or become loss to follow-up (LTFU) was also described.

We fitted different time-homogeneous multistate Markov models and observed that multistate models with both the orthogonal and the time-varying variables were the best fitting models to describe the HIV disease progression. The VL multistate model was superior in predicting the HIV disease progression patterns in this cohort compared to the CD4 cell count multistate model. These findings support what earlier studies have reported on the superiority of VL in monitoring HIV disease progression patterns compared to CD4 cell counts [7, 18]. Our findings further support the global guidelines on the use of VL as the primary laboratory marker to routinely monitor HIV disease progression amongst ART patients.

The VL multistate model with CD4 cell counts in the covariate matrix showed that VL rebound and VL increase transition rates (state 1 to 2; state 1 to 3 and state 2 to 3) were higher than the VL suppression transition rates (state 2 to 1; state 3 to 2 and state 3 to 1). This means viral rebound was more likely compared to viral suppression in this cohort. We also found that as the VL increased (*λ*_12_ and *λ*_23_) the transition rates increased with a decrease in the CD4 cell counts over time while as the VL decreased (*λ*_21_, *λ*_31_ and *λ*_32_) the transition rates decreased with a decrease in the CD4 cell counts decreased over time. This finding aligns with the negative correlation which exists between these two prognostic markers that as CD4 cell count levels decreases, VL increases. These finding could be explained by poor ART adherence which remains a challenge among ART patients [19]. Moreover, treatment failure or treatment side-effects may be possible underlying factors to explain these results [18]. With effective and potent ART; and without non-adherence challenges, the VL is expected to decrease as the CD4 cell counts increase. Therefore, interventions that support ART adherence like support groups, quick identification of treatment failure and continual education of the effectiveness of ART should be strengthened.

Becoming LTFU and mortality were more likely among patients with a VL ≥ 1000copies/uL (state 3 to 4 and state 3 to 5). Patients with CD4 cell counts lower than expected (negative orthogonal variable) were associated with an increased VL and mortality if VL > 50copies/uL. This means as patients with high VL immune deteriorate, the risk of death becomes higher. These findings support other earlier studies that once the immune system deteriorates, the chances of immune recovery become slim, and thus when most deaths occur [20–22]. The risk of becoming LTFU was high among patients with VL ≥ 50copies/uL and CD4 < 350cells/uL. This can be explained by the fact that more ill patients tend to drop out from the ART programme and are classified as LTFU [5]. However, some of these patients if tracked, would have died, which may result in a misclassification error of the LTFU outcome. In contrast, patients with higher CD4 cell counts were associated with becoming LTFU. This could be explained by the fact that much sicker patients are more likely to be bedridden; hence, they are hospital-bound while healthier patients may become LTFU as a result of “silent-transfers” to nearby health [23, 24].

Adjusting for the spatial covariate in the VL multistate model, as expected, the regions with a high risk of viral rebound were also more likely to have a high risk of low CD4 cell counts. The intrinsic MCAR prior model further confirmed a strong overlapping geographical correlation between individuals with a detectable VL (VL ≥ 50copies/uL) and immune deterioration (CD4 < 350cells/uL) of 66%; hence, a shared geographical pattern of relative risks between these two outcomes exists. Patients staying in provinces that border with nearby countries had high relative risks of immune deterioration and detectable VL, particularly, those from Matabeleland North province in the northern part of Zimbabwe. Matabeleland North province has a busy truck route to neighbouring countries and high mobility of local and tourists. Earlier studies looking at spatial heterogeneity of viral suppression in this province reported similar results [8, 25]. People in this province are likely to present late for health care [26], delay ART initiation [27] or engage in sexual activities with multiple partners which subsequently compromise viral suppression among HIV patients [28, 29]. Therefore, interventions such as self-testing [30], pre-exposure prophylaxis (PrEP) pills for high-risk groups [31] should be intensified in such regions.

The reported results should be inferred in light of some limitations. Firstly, the dataset used in this study is very small and might not be an accurate representation of the Zimbabwean HIV population. We included only health facility data liked to the ePMS with both VL and CD4 cell counts repeated measurements. In this regard, the MCAR assumption might not have been fully satisfied. The spatial effects were observed at a higher level which might not precisely pinpoint the marginalized areas to guide policy. This was as a result of our small sample size in this study; however, future studies should consider lower administrative level spatial units. Moreover, the MCAR model only describes the spatial interaction across the error terms to explain spatial autocorrelation; however, this model falls short when a more direct presentation of spatial interaction is desired. The VL and CD4 cell counts measurements were not randomly done rather differential monitoring was implemented due to resource constraints. The joint model could not account for the biological order of the association between VL and CD4 cell count, that is, VL change may precede CD4 change. We could not adjust for ART adherence, comorbidities (tuberculosis, diabetes and hypertension); and demographic characteristics (age and gender) due to model convergence issues. Multistate Markov models can estimate multiple transition rates and outcomes simultaneously compared to the Cox proportional hazard models. However, the assumption of constant hazard function does not reflect reality and also the Markov process has a memory loss property which may be a limitation in HIV studies [6]. Despite these limitations, this study managed to provide useful information in HIV monitoring through jointly modelling two HIV prognostic markers and identifying regions with poor immunological outcomes.

## Conclusion

In conclusion, the findings from this study provide a foundation on the HIV disease progression patterns in Zimbabwe to guide policy going forward and motivate future statistical modelling to consider such modelling approaches in infectious disease to guide policy and programme management. We found that VL models were much more superior compared to CD4 cell count models in HIV monitoring. We also observed that after joint modelling of CD4 cell counts and VL in a single model, the rates of VL rebound risks are still higher than viral suppression. We observed that having a high VL (VL ≥ 1000copies/uL) increased the risk of becoming LTFU and death. The spatial overlap between VL and CD4 cell counts indicates the inter-linkages between these two markers in HIV monitoring and the shared geographical correlation of HIV disease progression immunological outcomes was high. With the global efforts to achieve zero HIV incidence, interventions which encourage ART adherence like support groups and continual educational support on the barriers for ART uptake is crucial. Region-specific interventions which focus on behavioural change, social support, increased uptake of HIV preventions like PrEP should be strengthened among high-risk groups all in the quest to achieve zero HIV incidences by 2030.

## Supplementary Information

**Additional file 1.**

## Data Availability

The data that supports the findings of this study are available through an application process from the Ministry of Health and Child Care in Zimbabwe which is the custodian of the ePMS data through the AIDS/TB Unit who manages and oversees the ePMS data collection process and are the custodian of the database.
