# Supplementary material for: Markov modelling of viral load adjusting for CD4 orthogonal variable and multivariate conditional autoregressive mapping of the HIV immunological outcomes among ART patients in Zimbabwe
Source: Theor Biol Med Model. 2021 Aug 21;18:16. doi: 10.1186/s12976-021-00145-y (PMC8379787; doi:10.1186/s12976-021-00145-y)
Supplement: Supplementary file 1 — Additional file 1. [file 12976_2021_145_MOESM1_ESM.docx]

## *Appendix: The Multivariate Conditional Autoregressive (MCAR) model code*

model {

for (i in 1 : Nareas)

{

for (k in 1 : Ndiseases)

{

Y[i, k] ~ dpois(mu[i, k])

log(mu[i, k]) <- log(E[i, k]) + alpha[k] + S[k, i]

}

RR1[i] <- exp(alpha[1] + S[1, i])

RR2[i] <- exp(alpha[2] + S[2, i])

}

S[1:Ndiseases, 1 : Nareas] ~ mv.car(adj[], weights[], num[], omega[ , ])

for (i in 1:sumNumNeigh)

{weights[i] <- 1}

# Other priors

for (k in 1 : Ndiseases)

{alpha[k] ~ dflat()}

omega[1 : Ndiseases, 1 : Ndiseases] ~ dwish(R[ , ], Ndiseases)

sigma2[1 : Ndiseases, 1 : Ndiseases] <- inverse(omega[ , ])

sigma[1] <- sqrt(sigma2[1, 1])

sigma[2] <- sqrt(sigma2[2, 2])

corr <- sigma2[1, 2] / (sigma[1] * sigma[2])

mean1 <- mean(S[1,])

mean2 <- mean(S[2,])

}
